# Clinical analysis of fungal keratitis in patients with and without diabetes

**DOI:** 10.1371/journal.pone.0196741

**Published:** 2018-05-01

**Authors:** Jing Dan, Qingjun Zhou, Hualei Zhai, Jun Cheng, Lei Wan, Cheng Ge, Lixin Xie

**Affiliations:** 1 Department of Ophthalmology, Renmin Hospital of Wuhan University, Wuhan, Hubei Province, China; 2 Qingdao Eye Hospital, Shandong Eye Institute, Shandong Academy of Medical Sciences, Qingdao, Shandong Province, China; The Chinese University of Hong Kong, HONG KONG

## Abstract

We compared the clinical characteristics, treatments, and prognoses of fungal keratitis in patients with and without diabetes. Patients diagnosed with fungal keratitis at Shandong Eye Institute between January 2010 and December 2016 were retrospectively reviewed and classified as diabetic and nondiabetic groups. One-hundred-and-eleven patients (111 eyes) with diabetes and 740 patients (740 eyes) without diabetes were included. The diabetic patients showed significantly older (*p*< 0.05) and lower male:female ratio (*p*<0.05). Plants trauma was the primary risk factor in both groups, and there was no significant difference of pathogen type (the most common was *Fusarium* genus, followed by *Alternaria* and *Aspergillus* genera). Multivariate logistic regression analyses revealed that diabetes and topical glucocorticoid use were the independent risk factors for the severity of fungal keratitis. The recurrent infection rate between the diabetic and nondiabetic patients during the follow-up (6 to 24 months) after penetrating keratoplasty (PKP) was not significantly different. Although the recurrent epithelial defect, rejection, and best-corrected visual acuity were similar between the patients with matched bed/graft size (7.75/8.0 mm) in the two groups 1 year after PKP, the incidence of delayed re-epithelialization (>7 days) was significantly higher in diabetic patients (3/10 versus 2/43 in nondiabetic patients, *p*<0.05). More specially, the diabetic patients with the duration ≥10 years showed more significantly delayed re-epithelialization than those with the diabetic duration less than 10 years (3/5 versus 1/26, *p*<0.05). In conclusion, the diabetes mellitus is an independent risk factor that affect the severity of fungal keratitis. Corneal re-epithelialization was significantly delayed after PKP in the diabetic patients, especially with the duration ≥10 years.

## Introduction

Diabetes is one of the largest global health crises of this century. Patients with diabetes have an increased risk of fungal infection [1]. Fungal keratitis is a common sight-threatening infectious disease of the cornea, but there are few studies that have investigated fungal keratitis in patients with diabetes. Therefore, the aim of this study was to compare the epidemiological profiles, clinical characteristics, treatments, and prognoses of fungal keratitis in patients with and without diabetes.

## Materials and methods

### Patient selection

Patients with fungal keratitis, diagnosed by corneal scraping, culture, histology, or confocal microscopy, presenting at Shandong Eye Institute between January 2010 and December 2016, were identified by retrospectively reviewing medical records. The patients were categorized as with or without diabetes according to the 1999 WHO diabetes diagnostic criteria[2]. Patients were excluded if they had a malignant tumor or systemic disease requiring glucocorticoids or immunosuppressant medication. This study was approved by the institutional review board of Shandong Eye Institute and conformed to the guidelines of the Declaration of Helsinki, informed consent was waived because of the retrospective nature of the study and the data were analyzed anonymously.

### Demographic and baseline clinical data

Age, sex, time from onset of symptoms to hospital admission, pathogen type, antifungal drug sensitivity test result, treatment received, and prognosis were extracted from each patient’s medical record (S1 Dataset). For patients with diabetes, diabetes duration, Hemoglobin A1c (HbA1c), fasting plasma glucose (FPG), 2-hour postprandial glucose (2-h PG), and random plasma glucose were also recorded.

### Clinical manifestation and risk factors

Clinical manifestations, including initial best corrected visual acuity (BCVA), intraocular pressure, size and depth of corneal infiltration, hypopyon, perforation, endophthalmitis, and secondary glaucoma (>21 mmHg) were noted. Grading was conducted according to the method previously described by Hiraoka [3] and Prajna [4], with some modifications (Table 1). Total score reflects the severity of fungal infection (range, 0 to 12, ≤5 was defined as mild infection and ≥6 was defined as severe infection). Patients with positive culture results for the genera *Fusarium*, *Alternaria*, and *Aspergillus* were selected to explore correlations with age, diabetes status, topical glucocorticoid use, previous antifungal therapy, pathogen type, and the severity of fungal keratitis.

**Table 1:** Grading of corneal fungal infection.

Clinical manifestations	Score
Infiltrate^a^	
<2 mm	1
2–4 mm	2
>4 mm	3
% of Depth	
>0–33	1
>33–67	2
>67–100	3
Hypopyon	
Height <1 mm	1
1 mm < height <3 mm	2
3 mm < height	3
Perforation of cornea	1
Endophthalmitis	1
Secondary glaucoma	1

^a^Geometric mean of the longest diameter and longest perpendicular to that diameter in millimeters.

### Treatment and prognosis

Patients’ treatments were recorded and summed. Those with penetrating keratoplasty (PKP) were followed-up for 6 to 24 months, cases of fungal recurrence were recorded and compared between patients with and without diabetes. Among patients (of either group) who underwent PKP, the majority received bed/graft sized 7.75/8.00 mm, and the grafts were stored in the same preservation medium (DX medium), therefore, those patients were chosen to compare the corneal re-epithelialization time, recurrent epithelial defect, rejection, and BCVA at final follow-up (within 1 year) between the groups.

### Diabetic subgroups analyses

Patients with diabetes were divided into subgroups according to antidiabetic treatment history, metabolic control, and diabetes duration. Patients with a HbA1c level <7% were acknowledged as having well-controlled diabetes.

### Statistical analysis

Data analysis was conducted using SPSS software, version 19.0 (IBM SPSS Statistics, New York, USA). Quantitative variables were first assessed using Kologorov-Smirnov (1 sample) tests to assess normality of data distribution. Normally distributed data were expressed as mean and standard deviation (SD), and between-group comparisons were conducted using either Student’s t-tests or Welch’s t-tests. Data that were not normally distributed were expressed as medians and interquartile ranges (IQRs), and between-group comparisons were conducted using Mann-Whitney U tests. Qualitative variables were analyzed via Chi-square tests, continuity correction Chi-square tests, or Fisher’s exact tests. Multivariate logistic regression analyses were used to assess risk factors by adding significant forward substitution factors (according to univariate analyses). P-values <0.05 were considered statistically significant.

## Results

A total of 851 patients (851 eyes) were included in the study, 111 patients (111 eyes) with diabetes and 740 patients (740 eyes) without diabetes. There were more men than women in both groups, however, the male:female ratio was significantly different between the groups with and without diabetes (1.09:1 versus 1.85:1, respectively; *p*<0.05). The patients with diabetes were significantly older than the patients without diabetes (57.63±8.489 versus 54.73±1.795, respectively; *p*<0.05). There was no significant between-group difference in the time from symptoms onset to hospital admission (*p*>0.05). Risk factors did not differ significantly between the groups (*p*>0.05), cornea trauma (54.7%) was the predominant risk factor for fungal keratitis in all patients (particularly trauma from plants). No definite risk factors were appeared in 41.8% of patients, and only 3.5% of fungal keratitis cases were associated with ocular surface disease or surgery prior to presentation. Positive-culture eyes were similar between the groups with and without diabetes (53.2% versus 59.9%, respectively; *p*>0.05), and there was no statistical difference about the pathogen types between the two groups (the *Fusarium* genus was the most commonly isolated pathogen, followed by the *Alternaria* and *Aspergillus* genera, *p*>0.05). The *Candida* genus found in eyes of patients with versus without diabetes were similar (3.4% and 1.4%, respectively) (Table 2).

**Table 2:** Epidemiology and clinical profiles in diabetes and non-diabetes.

	Total (n = 851)	Diabetes (n = 111)	Non-diabetes (n = 740)	P value^a^
Sex No (%)				0.010
Male		58 (52.3)	480 (64.9)	
Female		53 (47.7)	260 (35.1)	
Male:Female		1.09:1	1.85:1	
Age (mean±SD) years		57.63±8.489	54.73±11.795	0.002^b^
From onset of symptoms to hospital admission Median (IQR) days		18 (10–30)	15 (9–30)	0.135^c^
Risk factors No (%)				0.457^d^
Plants trauma	256 (30.1)	35 (31.5)	221 (29.9)	
Other trauma^e^	209 (24.6)	21 (18.9)	188 (25.4)	
Ocular surface disease^f^	30 (3.5)	3 (2.7)	27 (3.6)	
No identified risk factor	356 (41.8)	52 (46.8)	304 (41.1)	
Pathogen No (%)		59 (53.2)	443 (59.9)	0.180
*Fusarium* genus	268 (53.4)	24 (40.7)	244 (55.1)	0.77^d^
*Alternaria* genus	117 (23.3)	18 (30.5)	99 (22.3)	
*Aspergillus* genus	77 (15.3)	13 (22.0)	64 (14.4)	
*Candida* genus	8 (1.6)	2 (3.4)	6 (1.4)	
Others	32 (6.4)	2 (3.4)	30 (6.8)	

Abbreviation:IQR, interquartile range

^a^Chi-square test

^b^Welch’s t test

^c^Mann-Whitney U test

^d^Fisher’s exact test

^e^Including dust, sand, metallic foreign body, glass, small winged insect, electric welding light, chemical material, sewage, contact lens, and unidentified foreign body.

^f^Including herpes simplx keratitis, and ocular surface surgery not long before presentation or who still needed to use topical glucocorticoid.

Drug sensitivity tests for the *Fusarium*, *Alternaria*, and *Aspergillus* genera (using itraconazole, fluconazole, voriconazole, and natamycin) revealed that the pathogens had the highest resistance to fluconazole (89.2%, 47.9%, and 51.9%, respectively). *Fusarium* was more resistant to itraconazole (65.7%) than natamycin (34.2%), *Aspergillus* was more resistant to natamycin (50.0%) than itraconazole (11.7%), and *Alternaria* was sensitive to itraconazole, voriconazole, and natamycin, but resistant to fluconazole (47.9%). All the three pathogens were sensitive to voriconazole (Table 3).

**Table 3:** Results of drug-resistant pathogens.

	Itraconazole	Fluconazole	Voriconazole	Natamycin^a^
Drug-resistant No (%)				
*Fusarium* genus (n = 268)	176 (65.7)	239 (89.2)	16 (6.0)	54 (34.2)
*Alternaria* genus (n = 117)	13 (11.1)	56 (47.9)	1 (0.9)	4 (7.8)
*Aspergillus* genus (n = 77)	9 (11.7)	40 (51.9)	0 (0)	20 (50.0)

^a^Some records of natamycin were missing, the existing datas of the three fungi above were 158, 51, and 40, respectively.

A total of 462 cases (462 eyes) with positive-culture of the *Fusarium*, *Alternaria*, or *Aspergillus* genera were chosen to ascertain independent risk factors for fungal keratitis severity. According to the clinical grading system (Table 1), 277 and 185 eyes were classified as mild and severe cases, respectively. Univariate analyses revealed that diabetes status, topical glucocorticoid use, and pathogen type were significantly different between the mild and severe groups (*p*<0.05) (Table 4). These three factors were further analyzed using multivariate logistic regression, diabetes status and topical glucocorticoid use were both independent risk factors for severe fungal keratitis (Table 5).

**Table 4:** Univariate analysis of risk factors for the severity of the clinical manifestation.

Factors	Mild (n = 277)	Severe (n = 185)	P value^a^
Age (mean±SD) years	53.26±12.144	55.16±10.868	0.086^b^
Diabetes status No (%)			0.008
Yes	24 (8.7)	31 (16.8)	
No	253 (91.3)	154 (83.2)	
Topical glucocorticoid use No (%)			0.036
Yes	10 (3.6)	15 (8.1)	
No	267 (96.4)	170 (91.9)	
Antifungal drugs No (%)			0.657
Yes	117 (42.2)	82 (44.3)	
No	160 (57.8)	103 (55.7)	
Pathogen No (%)			0.000
*Fusarium* genus	148 (53.4)	120 (64.9)	0.015
*Alternaria* genus	90 (32.5)	27 (14.6)	0.000
*Aspergillus* genus	39 (14.1)	38 (20.5)	0.068

^a^Chi-square test

^b^Student’s t test

**Table 5:** The multivariate logistic regression analysis.

Variable	B value	OR (95% Confidence Interval)	P value
Diabetes status	0.803	2.232 (1.255–3.969)	0.006
Topical glucocorticoid use	0.875	2.399 (1.047–5.499)	0.039
Pathogen	0.107	1.113 (0.864–1.433)	0.407

Among the 851 patients, 57 dropped-out of therapy (8 with and 49 without diabetes). Of the remaining 794 patients, 83.6% required surgical interventions. 27.2% and 9.2% of the 794 patients were treated with PKP and lamellar keratoplasty (LKP) respectively, and 3.0% lose their eyeballs. Treatments received by the groups with and without diabetes were similar (Table 6). The patients who received PKP were followed-up for 6 to 24 months, recurrence occurred in 2 cases (6.5%) with and 11 cases (5.9%) without diabetes, and the difference between the two groups was not significant (*p*>0.05). In 3 cases, recurrence occurred within 3 postoperative days, and in 10 cases, recurrence occurred within 2 weeks to 2 months. Ten cases with and 43 cases without diabetes were conducted using the same sized bed/graft (7.75/8.0 mm), with grafts stored in the same DX medium. Given that age and surgery duration may be associated with cornea re-epithelialization after PKP, we conducted between-group comparisons of these two factors, and found no significant differences (*p*>0.05). The incidence of delayed re-epithelialization (>7 days) was significantly different between the groups with and without diabetes (30% versus 4.7%, respectively; *p*<0.05) (Table 7), however, there were no significant differences between the two groups in terms of recurrent epithelial defect, rejection, and BCVA at final follow-up (within 1 year; *p*>0.05). All patients had better visual acuity than before surgery (Table 8).

**Table 6:** Treatment of the fungal keratitis.

	Diabetes (n = 103)	Non-diabetes (n = 691)	Total (n = 794)
Treatment No (%)			
PKP	31 (30.1)	185 (26.8)	216 (27.2)
LKP	5 (4.9)	68 (9.8)	73 (9.2)
Superficial keratectomy	36 (35.0)	268 (38.8)	304 (38.3)
Conjunctival flap	7 (6.8)	41 (5.9)	48 (6.0)
Drugs only	18 (17.5)	112 (16.2)	130 (16.4)
Evisceration/enucleation	6 (5.8)	17 (2.5)	23 (2.9)

**Table 7:** Cornea re-epithelialization after PKP.

	Diabetes (n = 10)	Non-diabetes (n = 43)	P value^a^
Age (mean±SD) years	56.70±12.248	51.37±8.732	0.114
Duration of surgery (mins)	47.00±8.233	47.26±7.777	0.926
Time of re-epithelialization No (%)			0.041^b^
≤7 days	7 (70)	41 (95.3)	
>7 days	3 (30)	2 (4.7)	

^a^Student’s t test

^b^Fisher’s exact test

**Table 8:** 1 year follow-up after PKP.

	Diabetes (n = 10)	Non-diabetes (n = 43)	P value^a^
Recurrent epithelial defect No (%)	1 (10)	2 (4.7)	0.473
Rejection No (%)	4 (40)	4 (9.3)	0.051^b^
BCVA before PKP (logMAR) Median (IQR)	2.04 (1.19–2.69)	2.39 (2.39–2.69)	0.261^c^
BCVA at last follow-up (logMAR) Median (IQR)	0.91 (0.49–1.57)	0.69 (0.52–1.00)	0.336^c^

Abbreviation:BCVA, best corrected visual acuity

^a^Fisher’s exact test

^b^Continuity correction Chi-square test

^c^Mann-Whitney U test

Among the 111 diabetic patients, 36 cases were diagnosed after admission, and among the 75 cases diagnosed before admission, only 39 cases had a history of antidiabetic drug treatments. There was no significant difference about the incidence of mild and severe fungal keratitis between the treated versus non-treated group (*p*>0.05) (Table 9). Glycemic control status (based on HbA1c) was not significant associated with the severity of fungal keratitis and the epithelial healing time postoperatively (including PKP and superficial keratectomy) (*p*>0.05). Diabetes duration was also not significant associated with the severity of fungal keratitis (*p*>0.05), however, the incidence of delayed re-epithelialization (>7 days) after PKP in patients with duration ≥10 years and <10 years were 60% (3/5) and 3.8% (1/26) respectively, the difference was significant (*p* = 0.008), and a statistical result closed to being significant after superficial keratectomy (*p* = 0.057) was obtained (Tables 9 and 10).

**Table 9:** Correlation between antidiabetic treatment history, glycemic control status, and diabetes duration with fungal keratitis severity.

	Mild (n = 57)	Severe (n = 54)	P value^a^
Antidiabetic treatments No (%)			0.433
Yes (n = 39)	22 (56.4)	17 (43.6)	
No (n = 72)	35 (48.6)	37 (51.4)	
Glycemic control status No (%)			0.335
HbA1c <7% (n = 16)	10 (62.5)	6 (37.5)	
HbA1c ≥7% (n = 95)	47 (49.5)	48 (50.5)	
Diabetes duration No (%)			0.902
<10 years (n = 92)	47 (51.1)	45 (48.9)	
≥10 years (n = 19)	10 (52.6)	9 (47.4)	

^a^Chi-square test

**Table 10:** Correlation between glycemic control status and diabetes duration with epithelial healing time postoperatively.

	Epithelial healing time postoperatively			
	PKP		Superficial keratectomy	
	≤7 days (n = 27)	>7 days (n = 4)	≤7 days (n = 25)	>7 days (n = 11)
Glycemic control status No (%)				
HbA1c <7.0%	2 (7.4)	0 (0.0)	4 (16.0)	2 (18.2)
HbA1c ≥7.0%	25 (92.6)	4 (100.0)	21 (84.0)	9 (81.8)
P value^a^	1.000		1.000	
Diabetes duration No (%)				
<10 years	25 (92.6)	1 (25.0)	23 (92.0)	7 (63.6)
≥10 years	2 (7.4)	3 (75.0)	2 (8.0)	4 (36.4)
P value^a^	0.008		0.057	

^a^Fisher’s exact test

## Discussion

Diabetes was reported as a major risk factor for fungal infection, and diabetic patients showed more severe clinical manifestations and worse prognoses after fungal infection [1,5]. In the present study, we compared the clinical characteristics, treatments, and prognoses of fungal keratitis in patients with and without diabetes. The results revealed that the diabetes mellitus is an independent risk factor that affect the severity of fungal keratitis. Corneal re-epithelialization was significantly delayed after PKP in the diabetic patients, especially with the duration ≥10 years.

Ocular trauma, ocular surface disease, and contact lens wear are the well-recognized risk factors for fungal keratitis. Here we found that there were no significant differences in the risk factors between the diabetic and nondiabetic group. Ocular trauma, especially from plants, was the primary risk factor for the fungal infection in the patients with or without diabetes, which is different with the contact lens wear as the primary risk factor of fungal keratitis in the USA [6]. There was only 1 case had a history of contact lens wear in our study. More diabetic patients (46.8% versus 41.1% without diabetes) had no identified risk factors, although the difference was not statistically significant, we can speculate this difference may be associated with diabetic keratopathy, which decreased the corneal sensation and caused the inability to sense the foreign bodies or trauma to the cornea. Moreover, there was no significant difference of pathogen type between the diabetic and nondiabetic group. The *Fusarium* genus (53.4%) was the most commonly isolated pathogen type, followed by the *Alternaria* (23.3%) and *Aspergillus* (15.3%) genera. The results were different from our study approximately 11 years ago which reported that the primary three pathogens were *Fusarium* (73.3%), *Aspergillus* (12.1%), and *Alternaria* (3.2%) [7], now the *Alternaria* genus has become the second most commonly isolated pathogen.

Diabetes is associated with a higher incidence of fungal infection[1]. Here we confirmed that diabetes and topical glucocorticoid application were two independent risk factors for the severity of fungal keratitis, but age, pathogen type, and previous use of anti-fungal drugs had no direct correlation with the severity. Mechanistically, long-term hyperglycemia may change the microenvironment of ocular surface, such as the tear components, ocular commensal, and the enzyme activity, which renders the fungal adherence, proliferation, and deep-layer penetration easier[8]. Moreover, compromised immunity associated with diabetes may decrease resistance to fungi. Treatment via corticosteroids is definitely contraindicated, as fungi replicate more freely in the presence of corticosteroids [9]. The growth patterns of fungal pathogens in the cornea are different, and sometimes associated with treatment options and prognoses, most *Fusarium* and *Alternaria* grow horizontally, while most *Aspergillus* grow vertically, in the cornea [10]. Univariate analyses revealed that *Aspergillus* occurred in similar proportions, while the proportions of *Fusarium* and *Alternaria* differed significantly between the mild and severe group. This may explain why, in our study, different pathogens showed no direct correlation with the severity of fungal keratitis. In this study, 43.1% (199/462) of patients had already been treated with topical or systemic antifungal drugs, including fluconazole and natamycin. The drug sensitivity tests showed drug resistant rates of *Fusarium*, *Alternaria*, and *Aspergillus* to fluconazole (89.2%, 47.9%, and 51.9%, respectively) and natamycin (34.2%, 7.8%, and 50.0%, respectively), so we think the increased resistance to conventional antifungal drugs may be associated with the poor therapeutic outcomes.

Penetrating keratoplasty is the only option if the fungal infection reaches the endothelium. Among the diabetic and nondiabetic patients with the matched size of bed/graft (7.75/8.0 mm), corneal re-epithelialization time were significantly different (30% versus 4.2%, respectively; *p<*0.05). This is consistent with the previous description [11]. There were no significant differences of recurrent epithelial defect, rejection, and BCVA at the final follow-up between the two groups. Among all patients who received PKP, no significant difference was noted in terms of fungal recurrence (6.5% versus 5.9%, respectively; *p*>0.05). Three cases recurred within 3 postoperative days, possibly due to incomplete lesion removal, the remaining 10 cases recurred between 2 weeks and 2 months, possibly due to the use of topical glucocorticoid.

In diabetic subgroups analyses, the severity of fungal keratitis was not significantly different between treated and non-treated diabetic patiens, this might be explained by the reason that most of the patients underwent antidiabetic treatments had not received standardized treatments and regular medical examination, glycemic control was not satisfactory. After admission, fasting blood-glucose (FBG) and 2-h PG were monitored. Patients needed operation intervention were required to control FBG 6-8mmol/L, and 2-h PG *<*11.1mmol/L.

The HbA1c level and diabetes duration were the common risk factors that influence the severity of various diabetic complications[12–14]. Among the diabetic changes in cornea, impaired epithelial regeneration caused by the long-term effects of hyperglycemia were reported as the major characteristics of diabetic keratopathy [15,16]. In our study, the severity of fungal ketatitis and epithelial healing time postoperatively were not significantly different between the well-controlled and the poorly-controlled diabetic patients. However, the diabetic patients with the duration ≥10 years showed significant higher incidence of delayed re-epithelialization after PKP (3/5 versus 1/26 in the patients with the duration less than 10 years, *p*<0.05). The results suggest that the diabetic duration, but not the HbA1c level, may be a more important risk factor of the delayed re-epithelialization in the diabetic patients with fungal infection after PKP. It should be mentioned that the small samples of diabetic patients were enrolled with the well-controlled hyperglycemia (16 cases) or duration ≥10 years (19 cases). Therefore, more cases are needed to analyze the correlation of HbA1c level and duration with severity of fungal keratitis in diabetic patients.

## Conclusions

Diabetes is an independent risk factor for the severity of fungal keratitis. Corneal re-epithelialization tends to be delayed in the diabetic patients after PKP, especially those with diabetes duration ≥10 years.

## Supporting information

S1 Dataset Dataset for the study. Clinical data for all the patients.
(XLS)
